#  Occurrence of Metopism in Dry Crania of Adult Brazilians

**DOI:** 10.5402/2013/158341

**Published:** 2013-08-04

**Authors:** Ivan do Nascimento da Silva, Katharina Jucá de Moraes Fernandes, Antônio José Casado Ramalho, Rodrigo Freitas Monte Bispo, Célio Fernando de Sousa Rodrigues, José Aderval Aragão

**Affiliations:** ^1^Faculdade Integrada Tiradentes (FITS), Avenida Comendador Gustavo Paiva 5017, Cruz das Almas, 57038-000 Maceió, AL, Brazil; ^2^Centro Universitário (Cesmac), Rua Cônego Machado, 918 Farol, 57051-160 Maceió, AL, Brazil; ^3^Universidade Federal de Alagoas, ICBS/UFAL, Praça Afrânio Jorge s/n, Prado, 57010-020 Maceió, AL, Brazil; ^4^Departamento de Morfologia, Cidade Universitária Professor José Aloísio de Campos, Avenida Marechal Rondon, s/n Jardim Rosa Elze, 49100-000 São Cristovão, SE, Brazil

## Abstract

The metopic suture is located between the tubercles of the frontal bone. There are divergences regarding the exact time at which it closes, which ranges from the first to the tenth year of life, although it may persist into adulthood. This study was conducted on 134 dry crania from adult Brazilians, of which 95 were male and 39 were female. These were available in the anatomy laboratories of higher education institutions in Maceió, AL, Brazil. All the crania were examined macroscopically with regard to the presence (metopism: M) on absence of the metopic suture. M was considered to be complete (Mc) when it continued uninterruptedly from the nasium to the bregma and incomplete (Mi) when it was not present over its entire length. It was observed that Mc was present in 4.48% (6/134) of the skull examined, of which 50% (3/134) were male and 50% (3/134) were female. An incomplete metopic suture was found in 5.22% (7/144) of the crania and more frequently among males (3.73%; 5/134). Among the crania with a metopic suture, the dolichocephalic type predominated (7.46%) in relation to brachycephalic crania (1.49%) and mesocephalic crania (0.74%). There was no predominance of metopism between the sexes, while an incomplete metopic suture was slightly more common among males.

## 1. Introduction

The frontal bone is a singular, median, and symmetrical bone that occupies the most anterior part of the cranium. It has joints with the parietal, ethmoid, sphenoid, nasal, zygomatic, lacrimal, and maxillary bones, thereby contributing towards uniting the neurocranium and the viscerocranium [1]. 

During the development stage, the frontal bone is a double bone, with right and left halves that grow together [2] and unite along the median line at the metopic suture [3]. This usually starts to undergo the fusion process at the age of two years [4] and may have completely fused by the age of six years [2, 5], eight years [4, 6], or ten years [1]. However, in approximately 8% of adults, the two halves of the frontal bone do not fuse [4], and the metopic suture persists. The suture may be incomplete or complete (when it extends from the nasium to the bregma), and this condition is known as metopism [7–9]. This name originates from the Greek word metopion, which means a space between the eyebrows [10].

Metopism has various degrees of incidence [11]: from 7%-8% among Europeans to 1% in Africans and 4-5% in Mongolians. Overall, the range of incidence can go from 1% to 12%, and it is slightly more prevalent among males [12].

The incidence of metopism and the difference in forms also vary according to geographical region [13]. In Lebanon it was 0.82% [14], in Nigeria 3.14% [13], in India 2.66% [15], in southern India 6.25% [16], and in Europe 7 to 10% [12, 17]. 

Prevalence of metopism is important, because, if not, this can be confused with a fracture of the frontal bone or even with a sagittal suture on radiological images [13, 18]. This is also important in paleodemography and forensic medicine [19]. In the present study, our objective was to determine the rate of occurrence of metopism in dry crania of adult humans.

## 2. Material and Method

134 dry crania from adult humans (95 from males and 39 from females) were analyzed. All of them belonged to anatomy laboratories in higher education institutions in Maceió, AL, and had been obtained in accordance with Law 8501 of November 30, 1992, which deals with use of unclaimed cadavers for teaching and research purposes. 

All the crania were macroscopically examined with regard to the presence or absence of the metopic suture. It was considered to be complete when it continued uninterruptedly from the nasium to the bregma and incomplete when it was not present over this entire length. 

The crania were also classified according to the cephalic index (CI) as follows: brachycephalic (CI > 80), dolichocephalic (CI < 80), or mesocephalic (CI = 80). This index was calculated by dividing the maximum width, that is, the distance between the euryons (the widest cross-sectional distance from side to side), by the maximum length, that is, the distance between the glabella and the opisthocranion (the glabella is located on the median plane, between the eyebrow arches, and the opisthocranion is the posterior point which is furthest from the longitudinal plane), multiplied by 100. The measurements of the maximum length and width of the cranium were made using Willis compasses. The crania were classified in accordance with Latarjet and Liard [1]. 

## 3. Results

**Figure 1:**
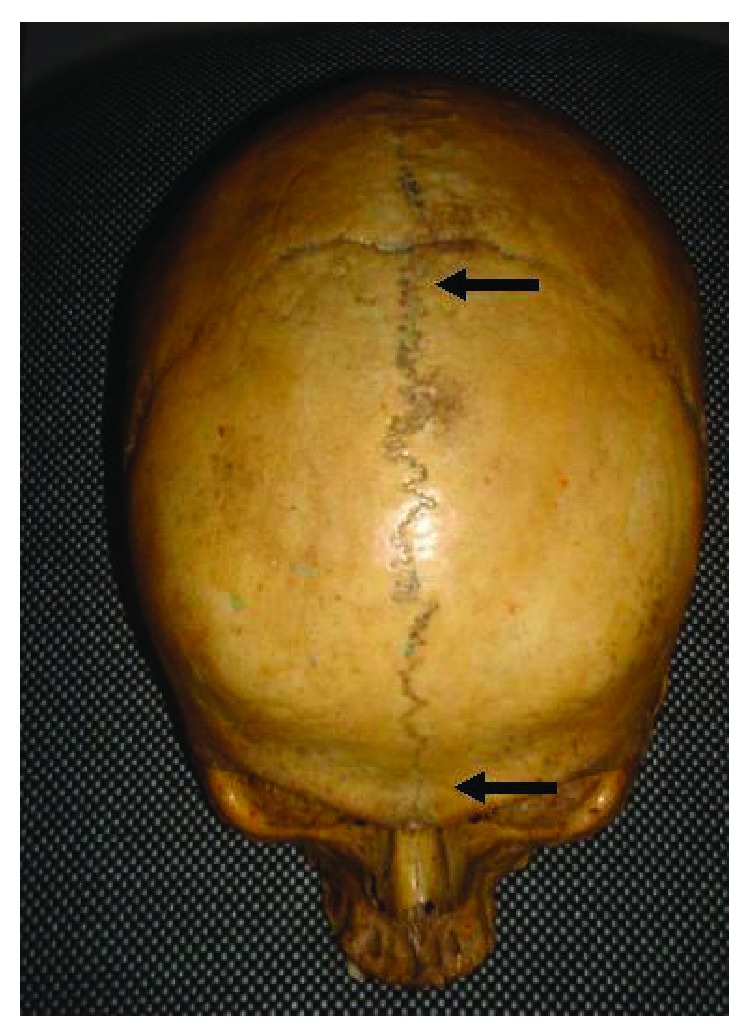
Complete metopic suture (arrow).

**Figure 2:**
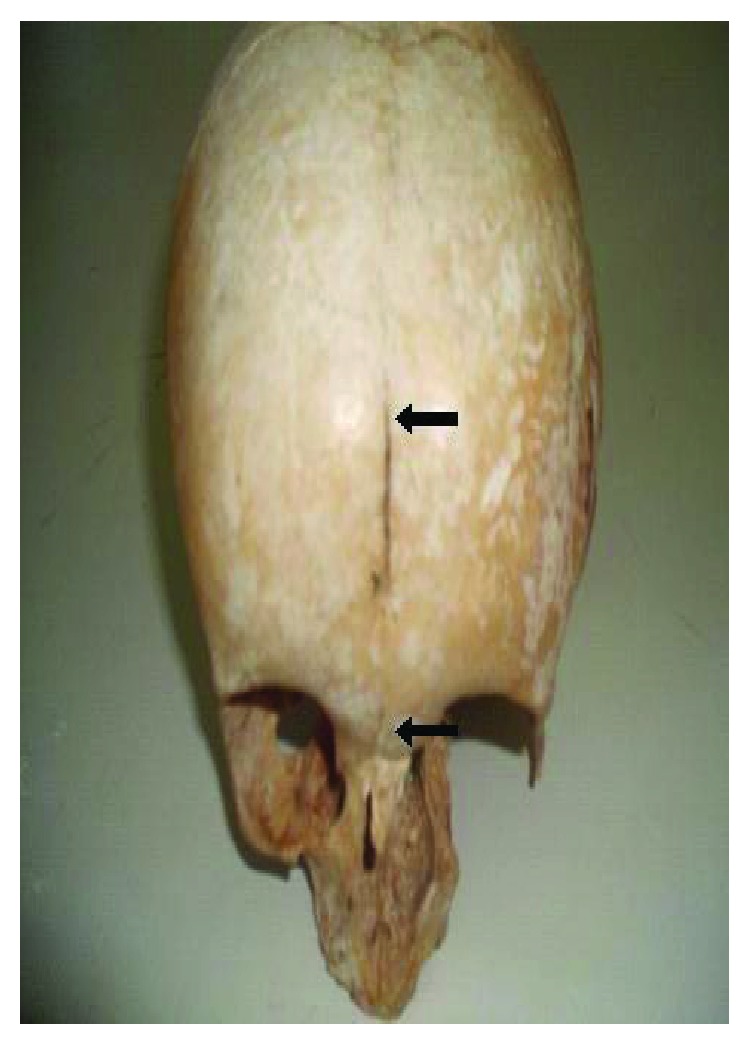
Incomplete metopic suture (arrow).

Out of the 134 crania examined, the metopic suture was found in 9.7% (13/134) of the cases, of which 61.5% (8/13) were in males and 38.5% (5/13) in females. A complete metopic suture (Figure 1) was found in six cases (46.2%), while an incomplete suture (Figure 2) was observed in seven cases (53.8%) (Table 1). 

According to the anthropometric characteristics of the crania studied by means of the cephalic index, 47.76% (64) were of brachycephalic type, 44.03% (59) were dolichocephalic, and 8.21% (11) were mesocephalic. However, when we classified the types of cranium according to whether a metopic suture was present, 7.46% (10) were dolichocephalic, 1.49% (2) were brachycephalic, and 0.74% (1) were mesocephalic. 

## 4. Discussion

There are divergences in the scientific literature in relation to the exact timing of closure of the metopic suture. Fusion has been reported as occurring between the first year and the beginning of the second year of life [28], before the sixth year [5], in the eighth year [4, 6], or in the tenth year [1]. According to Sant'Ana Castilho et al. [28], there are several causes of failure of the metopic suture to fuse, but genetic influence is currently the factor most accepted by the scientific community.

In the present study, metopism was found in 4.48% of the crania, which was similar to what was found in Indian populations (Punjab, Uttar Pradesh, and southern India), Mongolians, East Asians, and Nigerians (3.31 to 5.1%) but was lower than what was found among Scots, Mongoloids, and Europeans (7% to 10%) (Table 2).

A study conducted in Brazil by del Sol et al. [18] on 400 crania at the São Paulo Medical School (Escola Paulista de Medicina), Federal University of São Paulo, reported that the incidence of metopism was 2.75%. However, in an analysis on 71 crania at Paranaense University, Sant'Ana Castilho et al. [28] found an incidence of 7%. In the present study, out of the 134 crania found in anatomy laboratories in higher education institutions in Maceió, AL, the rate of occurrence of metopism was 4.48% and suture metopic 9.7%. This rate was higher than that found by either of the previous Brazilian studies. 

Among the 13 crania in which a metopic suture was seen to be present, the rate of occurrence was greater among males (61.54%), which corroborates the findings from studies by Skrzat et al. [12], Murlimanju et al. [29], and van der Meulen [30], in which there was also greater occurrence among males. However, this was contrary to the findings of Sant'Ana Castilho et al. [28], who observed that 80% of the occurrences of metopic suture were in females. According to Baaten et al. [14] the rates of occurrence were similar among males and females. 

However, when we correlated occurrences of metopic suture with the type of cranium, according to the cephalic index, we found that the occurrences in the dolichocephalic type were much greater than in the brachycephalic type. This differed from the findings of Sant'Ana Castilho et al. [28], who reported greater occurrence in the brachycephalic type. On the other hand, according to Bryce [31], what exists in reality are equal occurrences between the dolichocephalic and brachycephalic cranial types.

## 5. Conclusion

The rate of occurrence of metopism in dry crania in the city of Maceió was 4.48%, without predominance in one sex over the other. Incomplete metopic sutures were observed in 5.22% of the crania, with slight predominance among males. 

Knowledge of the possible presence of metopism serves to alert imaging professionals to the fact that this is just a variation and does not imply any harm to the population's health. Moreover, this variation should not be confused with a line of fractures in the frontal bone, especially if observed close to the median line.

## Figures and Tables

**Table 1:** Occurrence of types of metopic suture according to sex.

Types of metopic suture	Sex					
	Male		Female		Total	
	*n*	%	*n*	%		
Complete (metopism)	3	23.08	3	23.08	6	46.15
Incomplete	5	38.46	2	15.38	7	53.85

Total	8	61.54	5	38.46	13	100.00

**Table 2:** Incidence of metopism in different ethnic groups, as reported by various authors.

Study	Ethnic group	Occurrence (%)	Year
Jit and Shah [20]	Indian (Punjab)	5	1948
Das et al. [21]	Indian (Uttar Pradesh)	3.31	1973
Agarwal et al. [15]	Indian (Kanpur)	2.66	1979
Bryce [22]	European Mongolian Black African Australian Scottish	8.70 5.10 1.20 1.00 9.50	1917
Keith [23]	Subject to race	3–8	1948
WOO [24]	Mongoloid Negroid	10.0 2.00	1949
Frazer and Breathnach [17]	European East Asian African	7–10 4-5 1.00	1965
Romanes [25]	European	Up to 8.00	1972
Berry [26]	Various ethnic groups	7.40	1975
Ajmani et al. [13]	Nigerian	3-4	1983
Chandrasekaran and Shastri [27]	Indian (southern India)	5.00	2012
Present study	Brazilian (Maceió)	4.48	2013
